# Assessing quality of family planning counseling and its determinants in Kenya: Analysis of health facility exit interviews

**DOI:** 10.1371/journal.pone.0256295

**Published:** 2021-09-10

**Authors:** Susan Ontiri, Mark Kabue, Regien Biesma, Jelle Stekelenburg, Peter Gichangi

**Affiliations:** 1 Department of Health Sciences/Global Health Unit, University Medical Center Groningen, Groningen, The Netherlands; 2 Department of Monitoring Evaluation and Research, Jhpiego, Johns Hopkins University Affiliate, Nairobi, Kenya; 3 Department of Obstetrics and Gynecology, Leeuwarden Medical Centre, Leeuwarden, The Netherlands; 4 Department of Research, Technical University of Mombasa, Mombasa, Kenya; 5 Department of Public Health and Primary Care, Faculty of Medicine and Health Sciences, Ghent University, Belgium; 6 International Centre for Reproductive Health Kenya (ICRH-K), Mombasa, Kenya; Johns Hopkins Bloomberg School of Public Health, UNITED STATES

## Abstract

**Background:**

Available evidence suggests that provision of quality of care in family planning services is crucial to increasing uptake and continuation of use of contraception. Kenya achieved a modern contraceptive prevalence rate of 60% in 2018, surpassing its 2020 target of 58%. With the high prevalence, focus is geared towards improved quality of family planning services. The objective of this study is to examine the quality of family planning counseling and its associated factors in health facilities in Kenya.

**Methods:**

We conducted a secondary analysis of the 2019 Kenya Performance Monitoring and Action, client exit data of women who had received family planning services. Quality of counseling was assessed using the Method Information Index Plus. We conducted a multivariable ordinal logistic regression analysis of data from 3,731 women to establish determinants of receiving quality family planning services.

**Results:**

The Method Information Index Plus score for higher-quality counseling was 56.7%, lower-quality counseling 32.4%, and no counseling 10.9%. Women aged 15–24 years (aOR = 0.69, 95% CI = 0.56–0.86, p = 0.001) had lower odds of receiving better counseling compared to women aged 35 years and above. Those with no education (aOR = 0.52, 95% CI = 0.33–0.82, p = 0.005), primary (aOR = 0.56, 95% CI = 0.44–0.71, p<0.001) and secondary (aOR = 0.79, 95% CI = 0.65–0.98, p = 0.028) were less likely to receive better counseling compared to those with tertiary education. Women who received long acting and reversible contraception methods (aOR = 1.75, 95% CI = 1.42–2.17, p<0.001), and those who were method switchers (aOR = 1.24, 95% CI = 1.03–1.50, p = 0.027), had a higher likelihood of receiving better quality of counseling as compared to those on short-term methods and those who were continuers, respectively.

**Conclusion:**

The quality of family planning counseling in Kenya is still sub-optimal considering that some women receive no form of counseling at service delivery point. There is need to review the existing FP guidelines and training packages to increase focus on the quality of counseling services offered by health providers. Social accountability strategies that empower women to demand quality services should be included in community-level family planning interventions.

## Introduction

Addressing unmet need for contraception is a public health and development agenda espoused in several global commitments meant at supporting women to achieve their fertility aspirations. The sustainable development goal target 3.7 aim to ensure universal access to sexual and reproductive healthcare services, including family planning, (FP), information, and education by 2030 [1]. In 2019, the Nairobi Summit which marked the 25^th^ anniversary of the International Conference on Population and Development (ICPD) set an aspirational goal of zero unmet need for FP information and services and universal availability of quality, accessible, affordable, and safe modern contraceptives [2]. Unmet need for FP increases when contraceptive users discontinue, for reasons other than a reduced need for contraception [3, 4]. An analysis of demographic health survey (DHS) data from 34 developing countries found out that past contraceptive users with unmet need for modern methods accounted for 38% of all women with current unmet need [4]. In another DHS analysis from a diverse set of 15 countries, 7–27% of past contraceptive users discontinued due to reasons related to the quality of care they received [5]. Available evidence indicates that integrating quality of care in the provision of FP services at initiation is positively associated with the continuation of contraceptive use [5, 6], which then can reduce the unmet need for contraception. Other studies have also shown that providing high-quality FP services can lead to a higher level of satisfaction among users and thus increase continuous and consistent use among clients while they are in need of a method [7, 8].

Despite the existence of several tools that have been developed and used to measure the quality of care in FP, a global consensus on standardized measures for quality is still lacking [9]. Counseling, a key element in providing high-quality FP services, ensures that women are aware and understand what to expect when using a contraceptive method and how to handle health concerns and side effects which are the main reasons for discontinuation [6, 10, 11]. The Method Information Index (MII) is one recent approach created by Family Planning 2020 (FP2020) as a proxy indicator to track certain aspects of counseling, particularly information exchange between providers and clients and informed choice [12, 13]. The MII is a composite indicator that is calculated from responses to three questions that are asked to women about the information they received when they sought FP services. These questions seek to establish whether the women: were informed about methods other than the one they received, told about the method’s specific side effects, and advised on what to do if they experienced side effects. Participants who respond affirmatively to all three are assumed to have received quality counseling that encompasses the essential information clients need to choose a contraceptive method that meets their needs [14]. Although MII does not capture all aspects of FP counseling, it allows for monitoring of important components of information that women receive from their FP service providers [15]. MII is a simple measure, and can easily be integrated into the existing quality of care tools since all that is required is the inclusion of the three questions in the tools. Currently, MII has been included in the DHS as well as the Performance Monitoring for Action (PMA) survey on women’s questionnaire, to report on the quality of FP counseling at the national level [16].

A study that investigated the relationship between FP service quality and current contraceptive use among women in urban Kenya found out that indicators of FP service quality including discussing possible side effects with clients were significantly associated with current contraceptive use [17]. Findings from yet another study conducted in Uganda that utilized the MII to assess the risk of continuation over 12 months, revealed that women who reported being informed about all aspects of MII were 80% less likely to discontinue using contraception while in need as compared to women who reported not being informed about any aspect of MII [18].

In 2019, Jain et al. proposed a revised version of MII referred to MIIplus, which has a fourth question on whether women were informed of the possibility of switching to another method if the method selected was not suitable [13]. Jain et al. established that by adding the question about method switching to the MII, the MIIplus is a better predictor of continuation compared to the former [13]. Thus, providing information on method switching to women is an indication that the FP service delivery environment is supportive and responsive to women’s changing reproductive needs since women are aware they can switch to a method instead of abandoning the use of a contraceptive when they encounter challenges [19].

Concerted effort by the Government of Kenya and FP partners has led to an increase in the use of contraceptives. In 2018, Kenya achieved a modern contraceptive prevalence rate of 61% among married women, thus surpassing its target of 58% by the year 2020, with a notable increase in the use of implants at 38% [20, 21]. This increase in contraceptive use in Kenya should ideally translate to a reduction of unintended pregnancies, but that has not been the case; the rate of unintended pregnancies has remained unchanged over the last decade at 42% in both 2008–9 and 2018 national surveys [20, 21]. This could partly be attributed to the lack of consistent use of contraceptives among women in need. Indeed, one out of three women still discontinues the use of a contraceptive method within 12 months of initiation [20]. To date, much of the evidence on contraceptive discontinuation globally, including Kenya, shows that the majority of women abandoning use do so as a result of side effects and other health concerns. Despite this evidence on the reasons for contraceptive discontinuation, the 2014 Kenya DHS revealed that only 60% of contraceptive users were informed about potential side effects of their method, 52% were told what to do if they experienced side effects, and 79% were given information about other methods [20]. PMA 2019 survey further revealed that only 49% of women taking up FP services in Kenya received information on the four questions (MIIplus) from providers [22]. With Kenya having met the 2020 mCPR target, the government’s priority shifted to expanding equitable access to quality care at the county level rather than adjusting this target [23].

The 2012 London summit on FP bolstered global support and commitment towards increasing access to FP and reducing unmet need for contraception. Most countries including Kenya often rely on national surveys such as the DHS that are typically conducted every five years to provide estimates for FP data. Performance Monitoring and Accountability 2020 which later became Performance Monitoring for Action was created to provide rapid and frequent estimates of data on modern contraceptive use, demand, and quality in FP2020 priority countries including Kenya [24]. In this article, we report the results of a secondary analysis of PMA client exit data on the quality of FP counseling offered to clients and its associated determinants in Kenya.

## Methods

### Study design and settings

PMA collects nationally or a sub-nationally (county-level) representative sample of data from households and women in selected sentinel sites, to estimate FP and other health indicators annually. Regarding the design of the surveys, PMA uses a two-stage cluster design with typically urban-rural as the strata. A representative sample of enumeration areas (EAs) was drawn from a master sampling frame covered, provided by the Kenya National Bureau of Statistics (KNBS).

Service Delivery Points (SDPs) including all public health facilities, and up to three randomly chosen private facilities, whose catchment area was within the selected EA were included in the survey. The Service Delivery Points (SDPs), thus reflect the services available to a representative population, rather than being representative of all SDPs in the country. Details of the PMA methodology have been published elsewhere [24].

Data used for this analysis were based on the Kenya PMA client exit interview (CEI) cross-sectional survey that was conducted in the SDPs between November and December 2019 [25].

The study was conducted among a nationally representative sample of women from 11 out of 47 counties across Kenya, residing within 308 EAs. The PMA survey counties include Bungoma, Kericho, Kiambu, Kilifi, Kitui, Nairobi, Nandi, Nyamira, Siaya, Kakamega, and West Pokot. Based on the SDP’s average monthly client volume, a daily client volume was computed. Service Delivery Points were eligible to be included for CEI if their average client per day was three or more.

The data collection team comprised female research assistants who were either residents of the selected EA or resided close to the study EAs. They were trained on the tool and use of smartphone technology for data collection. All female clients who had received FP services on the day of the survey were approached for interviews as they exited the facilities. The questionnaire was administered to females aged 15 to 49 years once informed consent was obtained. Data were collected on socio-demographic characteristics of the respondents, experience of care, and client satisfaction with FP services that were provided. The final sample included 3930 women who completed the survey [25], of which 3731 women had complete data as per the PMA dataset (29 records that were dropped had incomplete data on all variables due to missing age values).

In this paper, analysis of the data conducted was further restricted to records of 3731 female clients who reported to either had received a modern contraceptive method namely, pills, injectables, condom, emergency contraceptive, implants, and intrauterine device (IUD), or a prescription for the same methods. Those who were further excluded from the analysis (n = 170) included; clients who did not receive contraceptive services (n = 155), those who reported having received male/female sterilization (n = 8) since the MIIplus questions on method switching would not be applicable, and those who received traditional methods and LAM (n = 7).

### Study variables

The outcome variable “quality of counseling received” was assessed using the MIIplus summary measure constructed from four questions: on whether during the visit the woman was 1) told about other FP methods, 2) informed about possible side effects or problems related, 3) told what to do if you experienced side effects or problems, and 4) told that you could switch to a different method if she wanted to. Responses to each of the four questions were coded as 0 (No) or 1 (Yes). A composite score was created as an overall sum of the four variables. Three categories of the quality of counseling received were identified—no counseling, lower-quality, and higher-quality. No counseling was defined as not being informed about any of the four questions- answering “no” to all the four questions. Lower-quality counseling was defined as being informed of either one, two, or three of the questions-where the respondents answered “yes” to either 1, 2, or 3 questions. Higher-quality counseling was defined as receiving all the information where respondents answered “yes” to all four questions. We compared the sensitivity of classifying the outcome into two categories versus four categories, and the results showed that there was no difference in the categorization.

The explanatory variables consisted of the characteristics of health facilities such as level of health facility (dispensary/health clinic, health center/nursing home, and hospital), and the managing authority (government or private). Variables on demographic characteristics of clients included age, which was coded into three categories, adolescents and youths 15–24 years, younger women 25–34 years, and older women 35 years and above; education level was coded into four categories, none, primary/vocational, secondary and tertiary; and marital status was coded into three categories, never married, formerly married (divorced/separated/widowed), currently married (currently married and living with a man). For wealth status, PMA uses the 10-step Economic Ladder Question (also known as the MacArthur ladder), which is a subjective tool where respondents rank themselves [26]. In this analysis, the wealth status variable was categorized into five strata, from lowest to highest. The type of contraceptive method received was coded as LARC (long-acting and reversible contraception) for implants and IUD, and short-term for injectables, pills, condoms, and emergency contraceptives. The type of user was coded into three categories based on the contraceptive used prior to their visit; adopters-those who reported not to have been using any method, continuers-those who were using the same method, and switchers- those who were using a different method from the one received on the day of the exit interview.

### Data analysis

Descriptive analyses were done which involved frequency tabulation of selected characteristics of the female contraceptive users. To establish the determinants influencing good quality of counseling, bivariate and multivariable ordinal logistic regressions were applied since our outcome variable was ordered from 0–no counseling, 1–lower-quality counseling, and 2–higher-quality counseling. Crude odds ratio (cOR), adjusted odds ratio (aOR), and 95% confidence intervals (CI) were used to measure the level of association between the explanatory and outcome variable from the regression analysis. We adopted a generalized linear model, customized for an ordinal outcome, accounting for clustering at health facility level in the multivariable analysis. A p-value of <0.05 was considered to be statistically significant; all variables were included in the final multivariable model. The analysis assumed proportional odds where the relationship between each pair of outcomes was the same; for example, no counseling versus lower-quality counseling or higher and higher-quality counseling versus lower or no-counseling. To test the proportional odds assumption, we used a likelihood ratio test that proved that the assumption holds. We had only one missing value for marital status which was excluded in the multivariable analysis.

### Ethical considerations

PMA data collection protocols were reviewed and approved by the Kenyatta National Hospital-University of Nairobi Ethics Research Committee and the Johns Hopkins Bloomberg School of Public Health Institutional Review Board. Additional ethical approval was not required since this study was based on secondary analyses of publicly available, deidentified data.

## Results

### Characteristics of female modern contraceptive users and quality of counseling

Table 1 shows that of the 3731 women who were included in the analysis, the majority resided in Nairobi County (17.4%) whereas West Pokot had the least representation of 1.3%. Only 1.5% visited a private facility while the remaining 98.5% received services from a government-owned health facility. Almost half of the respondents were aged 25–34 years (46.0%). Married women were the majority as they comprised 84.7% of the respondents. More than half of the respondents were continuers (56.8%). The proportion of women who received information on each of the aspects of MIIplus was more or less the same across the four components of the MIIplus, ranging from 71% to 79%. Using the MIIplus index, more than half of the women (56.7%) received higher-quality counseling, 32.4% received lower-quality counseling while 10.9% did not get any counseling. Further analysis of the MII score, which excludes the fourth question on method switching, revealed that 59.8% of the women reported having received information on all the three MII questions (S1 Table).

**Table 1:** Characteristics of female exit interview clients who received FP services.

	N	%
**County**		
Bungoma	476	12.8
Kericho	215	5.8
Kiambu	310	8.3
Kilifi	467	12.5
Kitui	283	7.6
Nairobi	651	17.4
Nandi	371	9.9
Nyamira	211	5.7
Siaya	256	6.9
Kakamega	441	11.8
West Pokot	50	1.3
**Type of facility**		
Hospital	987	26.5
Health center/Nursing/Maternity home	1270	34.0
Dispensary/Health clinic	1474	39.5
**Facility managing authority**		
Government	3676	98.5
Private	55	1.5
**Age (years)**		
15–24	1303	34.9
25–34	1715	46.0
35+	713	19.1
**Education level**		
None	88	2.3
Primary/Vocational	1664	44.6
Secondary	1297	34.8
Tertiary	682	18.3
**Marital status**		
Married	3160	84.7
Formerly married	201	5.4
Never married	369	9.9
Missing	1	0.0
**Wealth status**		
1-Lowest	451	12.1
2	1498	40.1
3	1496	40.1
4	237	6.4
5-Highest	49	1.3
**Contraceptive method received**		
Implant	1100	29.5
IUD	146	3.9
Injectables	1966	52.7
Pill	480	12.9
Condom	30	0.8
Emergency contraception	9	0.2
**User type**		
Adopter	681	18.3
Continuer	2120	56.8
Switcher	930	24.9
**MIIplus questions** *		
Informed about other FP methods	2755	73.8
Informed of possible side effects	2662	71.4
Informed of what to do in case of problems	2781	74.5
Informed about method switching	2934	78.6
**Quality of counseling (based on MIIplus)**		
No counseling	407	10.9
Lower-quality counseling	1208	32.4
Higher-quality counseling	2116	56.7
**Total**	**3731**	**100**

*The figures represents the number and percent of women reporting “Yes” to each of the four questions used as measures for the MIIplus indicator.

### Characteristics of women by quality of counseling

Table 2 shows characteristics of women by the quality of counseling. More than 50% of all women received higher-quality counseling in all the counties except Kilifi (49.9%) and Kakamega (49.4%). Almost three-quarters of the women from private facilities (74.5%) received higher-quality counseling. The proportion of women receiving higher-quality counseling increased with an increase in age 54.1% (15–24 years), 57.0% (25–34 years), 60.9% (35+ years). Similarly, the same trend of receiving higher-quality counseling was observed with an increase in education level-48.9% among those with no form of education, 58.9%-secondary level, and 66.1% among those with tertiary level of education. Among women with a LARC method, 66.3% received higher-quality counseling.

**Table 2:** Characteristics of female exit interview clients by quality of counseling received.

Variable	None		Lower-quality		Higher-quality	
	N	%	n	%	n	%
**County**						
Bungoma	67	14.1	132	27.7	277	58.2
Kericho	15	7.0	56	26.1	144	67.0
Kiambu	16	5.2	85	27.4	209	67.4
Kilifi	74	15.9	160	34.3	233	49.9
Kitui	19	6.7	62	21.9	202	71.4
Nairobi	68	10.5	236	36.3	347	53.3
Nandi	30	8.1	141	38.0	200	53.9
Nyamira	36	17.1	61	28.9	114	54.0
Siaya	33	12.9	82	32.0	141	55.1
Kakamega	48	10.9	175	39.7	218	49.4
West Pokot	1	2.0	18	36.0	31	62.0
**Type of Facility**						
Hospital	113	11.54	262	26.5	612	62.0
Health Center/Nursing home	154	12.1	470	37.0	646	50.9
Dispensary	140	9.5	476	32.3	858	58.2
**Managing authority**						
Government	407	11.1	1194	32.5	2075	56.4
Private	0	0	14	25.5	41	74.5
**Age**						
15–24	155	11.9	443	34.0	705	54.1
25–34	193	11.2	545	31.8	977	57.0
35+	59	8.3	220	30.8	434	60.9
**Education level**						
None	12	13.6	33	37.5	43	48.9
Primary	226	13.6	580	34.8	858	51.6
Secondary	127	9.8	406	31.3	764	58.9
Tertiary	41	6.2	189	27.7	451	66.1
**Marital Status**						
Married	350	11.1	1033	32.7	1777	56.2
Formerly married	29	14.4	61	30.4	111	55.2
Never married	28	7.6	113	30.6	228	61.8
**Wealth status**						
1-Lowest	65	14.4	173	38.4	213	47.2
2	161	10.8	494	33.0	843	56.3
3	159	10.6	465	31.1	872	58.3
4	19	8.0	61	25.7	157	66.2
5-Highest	3	6.1	15	30.6	31	63.3
**Type of contraceptive method**						
LARC	69	5.5	351	28.2	826	66.3
Short term methods	338	13.6	857	34.5	1290	51.9
**User type**						
Adopter	58	8.5	237	34.8	386	56.7
Continuer	280	13.2	692	32.6	1148	54.2
Switcher	69	7.4	279	30.0	582	62.6
**Total**	**407**	**10.9%**	**1208**	**32.4%**	**2116**	**56.7%**

### Determinants of quality of counseling

Findings from the multivariable analysis presented in Table 3 shows that women aged 15–24 years (aOR = 0.69, 95% CI = 0.56–0.86, p = 0.001), were less likely to receive better counseling compared to women aged 35+ years. Those with no education (aOR = 0.52, 95% CI = 0.33–0.82, p = 0.005), primary (aOR = 0.56, 95% CI = 0.44–0.71, p<0.001) and secondary (aOR = 0.79, 95% CI = 0.65–0.98, p = 0.028) were less likely to receive better counseling compared to those with tertiary education. Women who received LARC methods (aOR = 1.75, 95% CI = 1.42–2.17, p<0.001), and who were switchers (aOR = 1.24, 95% CI = 1.03–1.50, p = 0.027) had a higher likelihood of receiving better quality of counseling as compared to those on short-term methods and those who were continuers, respectively.

**Table 3:** Determinants of receiving better contraceptive counseling.

Variable	Bivariate			Multivariate		
	cOR	95% CI	p-value	AOR	95% CI	p-value
**County**						
Nairobi	Ref	Ref	Ref	Ref	Ref	Ref
Bungoma	1.11	0.58–2.14	0.755	1.01	0.51–2.00	0.979
Kakamega	0.88	0.51–1.52	0.642	0.89	0.52–1.54	0.681
Kericho	1.73	0.89–3.37	0.105	1.34	0.67–2.70	0.406
Kiambu	1.81	0.98–3.34	0.057	1.36	0.71–2.61	0.348
Kilifi	0.81	0.44–1.50	0.508	0.85	0.45–1.60	0.618
Kitui	2.10	1.06–4.17	**0.034**	1.94	0.97–3.91	0.063
Nandi	1.06	0.63–1.79	0.824	0.90	0.51–1.59	0.725
Nyamira	0.91	0.40–2.09	0.825	0.72	0.32–1.63	0.430
Siaya	1.02	0.56–1.84	0.956	0.87	0.47–1.61	0.664
West Pokot	1.54	0.68–3.48	0.303	1.33	0.63–2.85	0.454
**Type of Facility**						
Hospital	Ref	Ref	Ref	Ref	Ref	Ref
Health center	0.67	0.44–1.05	0.078	0.73	0.47–1.14	0.165
Dispensary	0.90	0.60–1.36	0.614	1.07	0.70–1.65	0.745
**Managing authority**						
Government	Ref	Ref	Ref	Ref	Ref	Ref
Private	2.42	1.25–4.67	**0.009**	2.02	0.96–4.25	0.064
**Age (years)**						
15–24	0.75	0.62–0.90	**0.003**	0.69	0.56–0.86	**0.001**
25–34	0.83	0.70–0.99	**0.040**	0.84	0.70–1.01	0.067
35+	Ref	Ref	Ref	Ref	Ref	Ref
**Education level**						
None	0.48	0.30–0.78	**0.003**	0.52	0.33–0.82	**0.005**
Primary	0.53	0.42–0.66	**<0.001**	0.56	0.44–0.71	**<0.001**
Secondary	0.72	0.59–0.88	**0.001**	0.79	0.65–0.98	**0.028**
Tertiary	Ref	Ref	Ref	Ref	Ref	Ref
**Marital Status**						
Married	1.10	0.80–1.50	0.563	1.09	0.80–1.48	0.583
Formerly married	Ref	Ref	Ref	Ref	Ref	Ref
Never married	1.41	0.97–2.06	0.075	1.34	0.92–1.96	0.129
**Wealth status**						
1-Lowest	0.51	0.24–1.07	0.075	0.68	0.35–1.32	0.259
2	0.72	0.35–1.50	0.385	0.87	0.46–1.66	0.678
3	0.78	0.38–1.60	0.496	0.79	0.42–1.49	0.469
4	1.09	0.51–2.33	0.820	1.04	0.53–2.04	0.920
5-Highest	Ref	Ref	Ref	Ref	Ref	Ref
**Type of contraceptive method**						
LARC	1.90	1.55–2.33	**<0.001**	1.75	1.42–2.17	**<0.001**
Short term	Ref	Ref	Ref	Ref	Ref	Ref
**Type of user**						
Adapter	1.18	0.96–1.44	0.113	1.05	0.85–1.30	0.66
Continuer	Ref	Ref	Ref	Ref	Ref	Ref
Switcher	1.48	1.22–1.79	**<0.001**	1.24	1.03–1.50	**0.027**

## Discussion

The purpose of our study that was conducted among exit clients who had received contraceptive services in public and private facilities in Kenya, was to assess the quality of FP counseling provided and to examine the predictors of the quality of FP counseling using the method information index plus. Our findings established that overall, 56.7% of women receiving FP services received higher-quality counseling, meaning that they were counseled on all four components of the MIIplus. To allow for comparison with other studies, we conducted additional analysis of the MII score (S1 Table), which revealed that 59.8% of women received information on all three questions (the fourth question on method switching question not included). In Uganda, an exit interview study conducted found out that 73.0% of women reported receiving information about all three MII aspects [18], higher than our results. However, the study in Uganda was limited to social franchise clinics hence was not indicative of public health facilities. A similar analysis of PMA CEI data in Ethiopia reported MII score of 30% [27]. The higher score established in our study compared to Ethiopia could be in part be explained by the efforts that Kenya has made in increasing access to modern contraceptive use, which has resulted in higher uptake. However, it is still a concern that almost two out of five women did not receive proper counseling, while 10.9% of the clients did not receive information on any of the four questions that comprises the MIIplus.

Our findings further revealed that younger women aged 15 to 24 years were less likely to receive better quality of counseling compared to older women aged 35 years and above. A possible explanation for this finding is that provider bias and preferences in the provision of contraceptive services to adolescents and youth has been shown to exist in Kenya [28], which might in part explain the poor quality of counseling received compared to older women. Furthermore, younger women are more likely to be initiating contraception compared to older women, thus our findings highlight a glaring gap in the quality of counseling offered. To support young women to consistently use contraceptives and thus reduce unmet need, it is important they are counseled on side effects, including options for method switching since evidence indicates that discontinuation among young women is higher compared to older women [29]. Moreover, younger women might want to try out several contraceptive methods as part of exploration before choosing a method that is best for them, which they will be using consistently. Thus, it is important that providers use the opportunities they have with the clients to share pertinent information on all contraceptive methods to leave room for potential method switching, as opposed to discontinuation.

The findings of this study further suggest that women with lower levels of education were more likely to receive lower-quality counseling compared to those with the highest level of education. These findings are similar to other study findings that have reported that women with higher levels of education are more likely to receive better counseling [27, 30]. These results could be an indication of a bias in the manner in which counseling is offered depending on one’s level of education. Alternatively, it could be mean that more educated women are likely to engage providers in conversation more and receive additional information as a result.

Our results further established that clients who chose LARC methods received better counseling compared to those who chose short-term methods. This is consistent with findings from other studies [27, 31]. Similarly, women who were switching contraceptive methods were more likely to receive higher-quality counseling compared to those who were continuers. There has been a deliberate effort to expand the method mix, particularly scale-up of implants in sub-Saharan Africa including Kenya [32], which may have resulted in switching behavior from short-term methods to LARC. Other studies have revealed that LARC methods are now being favored by health care providers based on efforts to scale up these methods [32, 33]. Whereas LARC methods have benefits including better continuation, good FP counseling ensures that all clients receive quality information; the level of counseling should not be dependent on the method a woman chooses. This is particularly important considering that short-term method users have higher rates of contraceptive discontinuation compared to users of LARC [11].

Our findings did not show any association between the level of counseling and the type of facility. This is contrary to findings from a similar study done in Ethiopia, which found out that those who got their method from the private sector had lower-quality of counseling [27]. However, a study conducted in Kenya reported that process quality was better at private facilities; 90% of clients from private health center facilities reported that providers asked about client concerns regarding methods or method use as compared to only 61% of providers at public health centers [34]. Our results may not have shown this association probably because of how the sampling of facilities was done, which limited inclusion of the number of private facilities in an enumeration area. Moreover, the threshold of serving a minimum of three clients in a day led to potential private facilities being excluded from the CEI survey. This could explain why in our analysis only 1.4% of the clients received services from private health facilities, which is not in line with the overall trends established in national surveys that reveal the private sector serve approximately 40% of FP clients [20].

Other studies have also established that quality of care is better at higher levels of facilities such as hospital. Our study results showed no significant association with the type of facility. This could be because, in Kenya, almost all contraceptive services are supposed to be available in all levels of facilities, from dispensaries to hospitals; this included implants which have increasingly become popular.

Surprisingly, our study did not find any difference in the level of counseling offered to adopters-women who were previously not using a method compared to continuers. With the high rate of discontinuation in Kenya of 31% [20], providing comprehensive information to women initiating use of a contraceptive should be paramount to achieve better continuation rates.

### Study limitations

The study findings should be interpreted in the context of the study’s limitations. First, the quantitative data were collected through client exit interviews, which rely on self-reporting and recall of the counseling session, hence may have been subject to recall bias. However, women were asked to recall a few minutes after receiving FP services, thus this short duration could potentially minimize recall bias. Secondly, MIIplus, which is a process indicator, does not fully capture all the components of quality of care. However, there is no global consensus on indicators that can measure the quality of FP that can be applied uniformly across the various health system [12]. Thus, the MIIplus remains one of the best available options that can inform on the quality of counseling offered to women. In addition, using the indicator allows data to be compared across different geographies since most countries have included it in their DHS. Thirdly, our regression analysis does not imply causation, but only an association between the explanatory and outcome variables. Lastly, an inherent limitation of client exit surveys is the convenience sampling approach that depends on the number of clients who come to the facility on the day of the survey, thus the sample is not chosen at random thus limiting the ability to make generalization beyond the sample.

## Conclusions

In conclusion, our findings show that reducing missed opportunities in providing adequate counseling to contraceptive users can promote informed choice. Our analysis has shown inequalities in provision of quality counseling that favors women with higher education levels compared to those with lower levels of education and older women aged 35 years and above compared to younger women aged 15–24 years. It is well documented that adolescent and youths have a higher unmet need for contraception of 23% compared to 18% for older women [20] which makes it crucial to ensure better quality of FP services is offered to these women, who also tend to have limited contraceptive access, to increase the use while in need.

Kenya’s high prevalence of modern contraceptive use means the country will likely experience slow growth in contraceptive uptake. Therefore, focusing on quality of care including counseling is essential to sustain the progress that has been achieved so far by ensuring women are given adequate information for better continuation and satisfaction with contraceptive methods.

There are programmatic implications that can be considered and incorporated into programs based on the findings of this study. Our overall findings indicate that a lot of effort is still needed to ensure all women receiving contraceptive services get high-quality counseling. Further, it underscores the importance of building the capacity of providers to offer adequate counseling to all contraceptive users, which is client-centered. Providers should be aware of the intentional or unintentional bias when offering contraceptive counseling to women, based on their age, education level, or contraceptive choice. Qualitative research among health care providers is needed to understand their perspectives, experiences, and barriers in providing quality contraceptive services to women. The Ministry of Health and partners should review its existing FP guidelines, training packages, and supportive supervision checklist to enhance focus on the quality of counseling services, including setting clear standards that health care providers can use. Social accountability strategies should be included in community-level FP interventions that empower women to demand quality services.

## Supporting information

S1 Table Quality of counseling based on the MII questions. (DOCX)

## References

[1] World Health Organization. Towards a monitoring framework with targets and indicators for the health goals of the post-2015 sustainable development goals. Geneva: World Health Organization. 2015.

[2] UNFPA. The Nairobi Summit Commitments on ICPD25 2019 [cited 2021 May 31]. Available from: https://www.nairobisummiticpd.org/content/icpd25-commitments.

[3] Barden-O’Fallon J, Speizer IS, Calhoun LM, Corroon M. Women’s contraceptive discontinuation and switching behavior in urban Senegal, 2010–2015. BMC women’s health. 2018;18(1):35. doi: 10.1186/s12905-018-0529-9 29402320 PMC5800088

[4] Jain AK, Obare F, RamaRao S, Askew I. Reducing unmet need by supporting women with met need. International Perspectives on Sexual and Reproductive Health. 2013;39(3):133. doi: 10.1363/3913313 24135045

[5] Blanc AK, Curtis SL, Croft TN. Monitoring contraceptive continuation: links to fertility outcomes and quality of care. Studies in family planning. 2002;33(2):127–40. doi: 10.1111/j.1728-4465.2002.00127.x 12132634

[6] RamaRao S, Mohanam R. The quality of family planning programs: concepts, measurements, interventions, and effects. Studies in family planning. 2003;34(4):227–48. doi: 10.1111/j.1728-4465.2003.00227.x 14758606

[7] Leisher SH, Sprockett A, Longfield K, Montagu D. Quality measurement in family planning: Past present future: Papers from the Bellagio Meeting on Family Planning Quality. Oakland, CA: Metrics for Management; 2015.

[8] Tessema GA, Streak Gomersall J, Mahmood MA, Laurence CO. Factors determining quality of care in family planning services in Africa: a systematic review of mixed evidence. PLoS One. 2016;11(11):e0165627. doi: 10.1371/journal.pone.0165627 27812124 PMC5094662

[9] Sprockett A. Review of quality assessment tools for family planning programmes in low-and middle-income countries. Health policy and planning. 2017;32(2):292–302. doi: 10.1093/heapol/czw123 28207050

[10] Lee JK, Parisi SM, Akers AY, Borrerro S, Schwarz EB. The impact of contraceptive counseling in primary care on contraceptive use. Journal of general internal medicine. 2011;26(7):731–6. doi: 10.1007/s11606-011-1647-3 21301983 PMC3138576

[11] Ali MM, Cleland JG, Shah IH, World Health Organization. Causes and consequences of contraceptive discontinuation: evidence from 60 demographic and health surveys. 2012.

[12] Jain AK, Townsend J, RamaRao S. Proposed metrics to measure quality: overview. 2018.

[13] Jain A, Aruldas K, Tobey E, Mozumdar A, Acharya R. Adding a question about method switching to the method information index is a better predictor of contraceptive continuation. Global Health: Science and Practice. 2019;7(2):289–99. doi: 10.9745/GHSP-D-19-00028 31249024 PMC6641810

[14] Chang KT, Mukanu M, Bellows B, Hameed W, Kalamar AM, Grépin KA, et al. Evaluating quality of contraceptive counseling: an analysis of the Method Information Index. Studies in family planning. 2019;50(1):25–42. doi: 10.1111/sifp.12081 30666641 PMC6590213

[15] RamaRao S, Jain AK. Aligning goals, intents, and performance indicators in family planning service delivery. Studies in family planning. 2015;46(1):97–104. doi: 10.1111/j.1728-4465.2015.00017.x 25753061

[16] Feeser K, Chakraborty NM, Calhoun L, Speizer IS. Measures of family planning service quality associated with contraceptive discontinuation: an analysis of Measurement, Learning & Evaluation (MLE) project data from urban Kenya. Gates open research. 2019;3. doi: 10.12688/gatesopenres.12974.2 32140663 PMC7042708

[17] Tumlinson K, Pence BW, Curtis SL, Marshall SW, Speizer IS. Quality of Care and Contraceptive Use in Urban Kenya. International perspectives on sexual and reproductive health. 2015;41(2):69–79. doi: 10.1363/4106915 . 26308259 PMC4548971

[18] Chakraborty NM, Chang K, Bellows B, Grépin KA, Hameed W, Kalamar A, et al. Association Between the Quality of Contraceptive Counseling and Method Continuation: Findings From a Prospective Cohort Study in Social Franchise Clinics in Pakistan and Uganda. Global Health: Science and Practice. 2019;7(1):87–102. 10.9745/GHSP-D-18-00407 PMC6538133 30846566

[19] Castle S, Askew I. Contraceptive discontinuation: reasons, challenges, and solutions. Population Council and FP2020. 2015.

[20] Kenya National Bureau of Statistics, Ministry of Health/Kenya, National AIDS Control Council/Kenya, Kenya Medical Research Institute, National Council for Population and Development, Kenya Medical Research Institute. Kenya Demographic and Health Survey 2014. Rockville, MD, USA: 2015.

[21] International Centre for Reproductive Health Kenya (ICRHK) and The Bill & Melinda Gates Institute for Population and Reproductive Health at The Johns Hopkins Bloomberg School of Public Health. Performance Monitoring and Accountability 2020 (PMA2020) Household and Female Survey Round 7, PMA2018/Kenya-R7-HQFQ. 2018. Kenya and Baltimore, Maryland, USA. 10.34976/p9dy-5h35.

[22] International Centre for Reproductive Health Kenya (ICRHK); The Bill & Melinda Gates Institute for Population and Reproductive Health at The Johns Hopkins Bloomberg School of Public Health; and Jhpiego. Performance Monitoring for Action (PMA) Client Exit Interview Phase 1, PMA2019/Kenya-P1-CQ. 2019. Kenya and Baltimore, Maryland, USA. 10.34976/5w8e-8512.

[23] FP2020. Commitments 2018 [cited 2021 May 3]. Available from: http://www.familyplanning2020.org/kenya.

[24] Zimmerman L, Olson H, Group PPI, Tsui A, Radloff S. PMA2020: rapid Turn‐Around survey data to monitor family planning service and practice in ten countries. Studies in family planning. 2017;48(3):293–303. doi: 10.1111/sifp.12031 28885679 PMC6084342

[25] International Centre for Reproductive Health Kenya (ICRHK) and The Bill & Melinda Gates Institute for Population and Reproductive Health at The Johns Hopkins Bloomberg School of Public Health. Performance Monitoring for Action (PMA) Kenya Phase 1: Service Delivery Point Client Exit Interview Baseline PMA/Kenya-P1-CQ-Baseline. 2019. Kenya and Baltimore, Maryland, USA.

[26] Amir D, Valeggia C, Srinivasan M, Sugiyama LS, Dunham Y. Measuring subjective social status in children of diverse societies. Plos one. 2019;14(12):e0226550. doi: 10.1371/journal.pone.0226550 31860691 PMC6924674

[27] Hrusa G, Spigt M, Dejene T, Shiferaw S. Quality of Family Planning Counseling in Ethiopia: Trends and determinants of information received by female modern contraceptive users, evidence from national survey data,(2014–2018). PloS one. 2020;15(2):e0228714. doi: 10.1371/journal.pone.0228714 32040485 PMC7010283

[28] Tumlinson K, Okigbo CC, Speizer IS. Provider barriers to family planning access in urban Kenya. Contraception. 2015;92(2):143–51. doi: 10.1016/j.contraception.2015.04.002 25869629 PMC4506861

[29] Blanc AK, Tsui AO, Croft TN, Trevitt JL. Patterns and trends in adolescents’ contraceptive use and discontinuation in developing countries and comparisons with adult women. International perspectives on sexual and reproductive health. 2009:63–71. doi: 10.1363/ipsrh.35.063.09 19620090

[30] Abdulreshid M, Dadi HB. Assessment of Family Planning Counseling Provided for Postpartum Women and Associated Factors. International Journal of Reproductive Medicine. 2020;2020. doi: 10.1155/2020/2649340 32047803 PMC7007746

[31] Jain AK. Examining progress and equity in information received by women using a modern method in 25 developing countries. International perspectives on sexual and reproductive health. 2016;42(3):131–40. doi: 10.1363/42e1616 28825904

[32] Jacobstein R. Liftoff: the blossoming of contraceptive implant use in Africa. Global Health: Science and Practice. 2018;6(1):17–39. doi: 10.9745/GHSP-D-17-00396 29559495 PMC5878070

[33] Teferra AS, Wondifraw AA. Determinants of long acting contraceptive use among reproductive age women in Ethiopia: evidence from EDHS 2011. Science Journal of Public Health. 2015;3(1):143–9.

[34] Track 2020. The S-Curve: Putting mCPR Growth into Context October 20172017 [cited 2019 03/05/2019]. Available from: http://www.track20.org/pages/data_analysis/in_depth/mCPR_growth/s_curve.php.

