# Supplementary material for: Assessing quality of family planning counseling and its determinants in Kenya: Analysis of health facility exit interviews
Source: PLoS One. 2021 Sep 10;16(9):e0256295. doi: 10.1371/journal.pone.0256295 (PMC8432739; doi:10.1371/journal.pone.0256295)
Supplement: S1 Table — (DOCX) [file pone.0256295.s001.docx]

S1 Table- Quality of Counseling based on the MII questions

| **Method information index** | **n** | **%** |
| --- | --- | --- |
| Not informed on any of the three questions 0/3 | 552 | 14.8 |
| Informed 1/3 questions | 392 | 10.5 |
| Informed 2/3 questions | 555 | 14.9 |
| Informed on all the 3 questions | 2232 | 59.8 |
